# Clinical and Translational Applications of Serological and Histopathological Biomarkers in Metastatic Breast Cancer: A Comprehensive Review

**DOI:** 10.3390/ijms24098396

**Published:** 2023-05-07

**Authors:** Leonel Pekarek, Alicia Sánchez Cendra, Eduardo D. Roberts Cervantes, Cristina Sánchez Cendra, Oscar Fraile-Martinez, Cielo García-Montero, Raul Diaz-Pedrero, Diego Torres-Carranza, Laura Lopez-Gonzalez, Soledad Aguado-Henche, Antonio Rios-Parra, Luis M. García-Puente, Natalio García-Honduvilla, Julia Bujan, Melchor Alvarez-Mon, Miguel A. Saez, Miguel A. Ortega

**Affiliations:** 1 Department of Medicine and Medical Specialities, Faculty of Medicine and Health Sciences, University of Alcalá, 28801 Alcala de Henares, Spain; 2 Ramón y Cajal Institute of Sanitary Research (IRYCIS), 28034 Madrid, Spain; 3 Oncology Service, Guadalajara University Hospital, 19002 Guadalajara, Spain; 4 Department of Surgery, Medical and Social Sciences, Faculty of Medicine and Health Sciences, University of Alcalá, 28801 Alcala de Henares, Spain; 5 Department of General and Digestive Surgery, General and Digestive Surgery, Príncipe de Asturias Universitary Hospital, 28805 Alcala de Henares, Spain; 6 Pathological Anatomy Service, University Hospital Príncipe de Asturias, 28806 Alcala de Henares, Spain; 7 Immune System Diseases-Rheumatology, Oncology Service an Internal Medicine (CIBEREHD), University Hospital Príncipe de Asturias, 28806 Alcala de Henares, Spain; 8 Pathological Anatomy Service, Central University Hospital of Defence-UAH Madrid, 28801 Alcala de Henares, Spain; 9 Cancer Registry and Pathology Department, Principe de Asturias University Hospital, 28806 Alcala de Henares, Spain

**Keywords:** metastatic breast cancer, serum biomarkers, histopathological biomarkers, MicroRNA, circulating tumor cells

## Abstract

Breast cancer is one of the most common malignancies worldwide and the most common form of cancer in women. A large proportion of patients begin with localized disease and undergo treatment with curative intent, while another large proportion of patients debuts with disseminated metastatic disease. In the last subgroup of patients, the prognosis in recent years has changed radically, given the existence of different targeted therapies thanks to the discovery of different biomarkers. Serological, histological, and genetic biomarkers have demonstrated their usefulness in the initial diagnosis, in the follow-up to detect relapses, to guide targeted treatment, and to stratify the prognosis of the most aggressive tumors in those with breast cancer. Molecular markers are currently the basis for the diagnosis of metastatic disease, given the wide variety of chemotherapy regions and existing therapies. These markers have been a real revolution in the therapeutic arsenal for breast cancer, and their diagnostic validity allows the classification of tumors with higher rates of relapse, aggressiveness, and mortality. In this sense, the existence of therapies targeting different molecular alterations causes a series of changes in tumor biology that can be assessed throughout the course of the disease to provide information on the underlying pathophysiology of metastatic disease, which allows us to broaden our knowledge of the different mechanisms of tissue invasion. Therefore, the aim of the present article is to review the clinical, diagnostic, predictive, prognostic utility and limitations of the main biomarkers available and under development in metastatic breast cancer.

## 1. Introduction. Epidemiology, Diagnosis, Molecular Classification, and Prognosis of Metastatic Breast Cancer

Breast cancer is the most common form of cancer in our population and the leading cause of cancer-related death in women in most developed and undeveloped countries. In 2022, approximately 3 million cases of breast cancer were diagnosed worldwide, and more than 600,000 deaths occurred in the same period, representing approximately 13% of all malignancies diagnosed worldwide [1]. In women, breast cancer accounts for a quarter of all neoplasms diagnosed, with an overall incidence of 50 cases per 100,000 women [2]. It should be noted that there are differences within developed countries with regard to incidence, with Belgium having the highest rate of diagnosis at 113 cases per 100,000 women [3]. In recent decades, there has been a 30–40% increase in the incidence of breast cancer due to the systematic implementation of mammography screening programs in at-risk age groups, which has allowed the detection of disease at earlier stages and has undoubtedly improved the prognosis [4]. Different risk factors have been described, of which several stand out. For example, age is a major risk factor for metastatic breast cancer (MBC). Women over 50 years of age are at higher risk than younger women. Studies have also found a higher incidence of MBC in post-menopausal women. A family history of breast cancer is another risk factor for MBC; women with a family history of breast cancer have an increased chance of developing it. Previous diagnosis of breast cancer is also an indicator of potential risk [5]. Women who have already been diagnosed with breast cancer are at higher risk for MBC. The risk increases depending on the stage of the initial cancer, the presence of metastasis, and the type of treatment received. Certain gene mutations, such as *BRCA1* and *BRCA2*, *TP53*, *CDH1*, *PTEN*, and *STK11*, can greatly increase the risk of MBC [6]. Mutations to these genes can be either inherited or acquired over a person’s lifetime. Another risk factor is obesity and being overweight which is associated with a greater risk of developing MBC, even in women with no family history of the disease, and it is related to a worse prognosis [7]. Studies have found a correlation between excessive and regular alcohol consumption and an increased risk of developing MBC. This association is especially true for women aged 55 and over [8]. Long-term use of hormone replacement therapies, such as birth control pills or hormone replacement therapy (HRT), has been associated with an increased risk of developing MBC [9]. These are just some of the risk factors associated with MBC. Today, the prognosis has undoubtedly improved following the implementation of screening programs. Breast cancer screening is important because it can detect cancer at an early stage when treatment is most effective. In this regard, the U.S. Preventive Services Task Force (USPSTF) has developed guidelines for breast cancer screening that are widely used by healthcare professionals. The 2021 guidelines recommend that women at average risk of breast cancer should begin mammography screening at age 50 and continue to be screened every two years until age 74 [10].

Diagnosis of breast cancer begins with a physical examination and imaging tests of the breast, such as a mammogram or ultrasound, to detect any suspicious lumps or areas of thickening. If a lump is found, the next step is usually a needle biopsy to obtain tissue samples from the area in question, which are examined microscopically for cancer cells [11]. Additional tests may include MRI (magnetic resonance imaging) for high-risk patients (such as *BRCA*-positive mutations), CT (computed tomography), or PET (positron emission tomography) scans to obtain additional information on the extent of the cancer and whether it has spread to other areas of the body [12]. All imaging tests allow stratification of the extent of the disease according to tumor size, lymphatic extension, and distant dissemination. Approximately 64% of women are diagnosed with local involvement, 27% with regional involvement (locoregional lymph node involvement), and 6% are diagnosed at an advanced stage. Regardless of the diagnostic staging, histological and pathological markers are essential to classify the type of neoplasm [13,14].

At present, the expression of hormone receptors and HER2/neu (also known as ErbB-2 tyrosine kinase) are used for molecular classification with different therapeutic and prognostic implications. The classification proposed by Kapp et al. explains that 71% represent the HER2-negative group with positive hormone receptors, while 12% are positive for both markers (HER2 and hormone receptors), another 12% are negative for both markers and are the worst prognostic group, and 5% are only positive for HER2 [15,16]. As with the diagnostic classification, the treatment of MBC can be divided according to the expression of different molecular markers, given the existence of targeted therapies. In HER2-negative metastatic tumors with positive hormone receptors, the first-line treatment is based on hormone blockade with aromatase inhibitors or an antiestrogen associated with a CDK 4/6 inhibitor, such as palbociclib. In triple-negative tumors, it is important to assess PD-L1 status in order to administer immunotherapy. In November 2020, the FDA granted accelerated approval to pembrolizumab, an anti-PD-L1 antibody, in combination with chemotherapy for the treatment of patients with metastatic TNBC whose tumors express PD-L1 (CPS ≥ 10), and in the case of null PD-L1 expression, therapy is based on chemotherapy, although with limited clinical benefit [17]. In addition, it is important to assess *BRCA1* and *BRCA2* expression in these patients, given the clinical benefit of administering poly ADP ribose polymerase (PARP) inhibitors as targeted therapy, which has changed the prognosis of these patients. On the other hand, in HER2-positive metastatic tumors, the first-line treatment is based on trastuzumab, pertuzumab, and chemotherapy with taxanes [18]. It should be noted that MBC presents numerous lines of treatment, highlighting the different clinical trials that are currently being carried out that demonstrate the usefulness of new therapies, such as the combination of conjugated antibodies with chemotherapy such as trastuzumab deruxtecan [19].

It is important to note that throughout metastatic disease, follow-up imaging tests such as CT or PET scans are complemented using serological tumor markers to detect elevations. Serological levels of tumor markers allow easy detection of possible tumor recurrence and modification of systemic treatment using a peripheral blood sample [20]. On the other hand, the importance of new biomarkers, such as circulating tumor cell microRNA and immunohistochemical and genetic markers in different tumors, has been demonstrated elsewhere, and their diagnostic and prognostic usefulness has also been evaluated in breast cancer. Therefore, we can see how different molecular markers in MBC are important from diagnosis to treatment and how different targeted therapies are being developed to broaden the therapeutic arsenal and improve the prognosis of patients with advanced breast cancer.

## 2. Serological Markers in Metastatic Breast Cancer

Over time, different biomarkers have been incorporated into the study and development of cancer treatments. These biomarkers have been gradually introduced into clinical practice at different levels of care (early detection and diagnosis, prognostic and predictive factors, etc.). They can be measured both in the tumor tissue itself and in biological samples. There are different detection techniques, such as immunohistochemistry, fluorescence in situ hybridization (FISH), or polymerase chain reaction (PCR), for sequencing genetic material. Nowadays, more precise and productive techniques, such as Next-Generation Sequencing (NGS), which performs an exhaustive analysis of the genetic material through the sequencing of multiple DNA fragments in parallel, are being used [21]. This technique has the limitation that it is not available in all centers. On the other hand, multigene panels such as Oncotype DX and MammaPrint have been commonly used to assess the risk of breast cancer recurrence and the benefits of targeted therapy [22]. In recent years, techniques such as liquid biopsy have been developed in which we can study, through a blood test, the presence or absence of certain genetic mutations in relation to a tumor process [23]. Mutations in either of the type 1 or 2 breast cancer susceptibility genes (*BRCA1* and *BRCA2*) account for the majority of hereditary breast cancers. Numerous mutations in these genes have been identified that affect proper DNA repair and cause irregularities in DNA synthesis [24].

In general, pathogenic variants in these genes are implicated in approximately 15 percent of women with familial breast cancer [25], with an autosomal dominant inheritance pattern, a particularly early disease onset, and a higher incidence of tumors of other organs, such as the fallopian tubes, prostate, or pancreas. Therefore, patients carrying these mutations should be offered surveillance with imaging studies or analytical controls from time to time [26].

Knowing whether patients are carriers of this mutation is predictive because therapies such as olaparib are approved by the Food and Drug Administration (FDA) for the treatment of patients with HR+/HER2− MBC with germline *BRCA* mutations [27].

As for tumor markers in breast cancer, those molecules that we can detect in blood and whose presence can guide us in the diagnosis of a given tumor have a sensitivity of 25–30% in locoregional tumors and 75–85% in metastatic tumors [26]. They are not very specific, so they are not useful for diagnosis or screening, and their main application is to monitor disease progression and/or response to a given treatment. All the biomarkers are summarized in Table 1.

### 2.1. Serological CA15-3. Most Clinical Marker Used in MBC

In many patients with breast tumors, the production of carbohydrate antigen 15-3 (CA 15-3) and carbohydrate antigen 27-29 (CA 27-29) is increased [28]. Therefore, guidelines from the American Society of Clinical Oncology (ASCO) expert panel suggest that it is reasonable to assess markers such as CA 15-3 and CA 27-29 initially in patients with metastatic disease. If CA 15-3 and/or CA 27-29 are elevated, there would be no need to monitor other markers, but if not, serial measurement of carcinoembryonic antigen (CEA) levels may be useful [29,30]. CA 15-3 is a protein naturally produced by breast cells; it binds to the high-molecular-weight DF3 antigen located at the apical border of breast epithelial cells. Its elevation varies according to the type of disease; therefore, its measurement is not useful in all cases. This marker is elevated in less than 50% of women with localized or early breast cancer but is elevated in 80% of cases of advanced breast cancer, and in some individuals, it is not detected at any stage of the disease [31,32]. CA 15-3 may be elevated in other cancers (colon, lung, pancreatic, ovarian, or prostate) and in other non-tumor situations (cirrhosis, hepatitis, and benign breast disease). The increase in CA 15-3, the first sign of tumor recurrence in 37% of patients with metastases, has been demonstrated by authors such as De Cock et al. in 730 patients, which shows its importance in the follow-up of these patients [33].

### 2.2. Serological CA 27-29. Supporting Serological Monitoring

In this sense, another of the markers that have gained importance in recent years is CA 27-29. CA 27-29, also known as MUC-1 (mucin 1), is a large transmembrane glycoprotein expressed on the surface of various epithelial cells. Overexpression of CA 27-29 has been observed in breast cancer cells, particularly in advanced stages of the disease. Elevated serum levels of CA 27-29 have been associated with tumor burden and metastatic spread in breast cancer patients [34]. Several studies have shown that CA 27-29 levels may be useful for monitoring disease progression in patients with MBC and have shown that an increase in CA 27-29 levels may precede radiological evidence of disease progression, suggesting that it may serve as an early indicator of treatment failure or tumor recurrence. In this regard, CA 27-29 levels have been found to correlate with treatment response in patients with MBC. For example, a decrease in CA 27-29 levels after systemic treatment, such as chemotherapy or targeted therapy, may indicate a favorable response, while persistently elevated or rising levels may suggest resistance or lack of response to treatment [35]. On the other hand, elevated CA 27-29 levels have been associated with a worse prognosis in patients with MBC. Higher baseline CA 27-29 levels may predict shorter progression-free survival and overall survival in these patients, underlining its potential as a prognostic marker [36].

Although serological markers offer a non-invasive approach to monitoring MBC, their diagnostic and prognostic utility as stand-alone markers remains limited. The low sensitivity and specificity of these markers can lead to false-positive or false-negative results. Combining several markers or incorporating them into a panel with other diagnostic tools, such as imaging and histopathology, may increase their clinical utility. In conclusion, further research is needed to identify new serological markers and explore their potential in personalized medicine. The integration of serological markers with tumor molecular profiling and advances in liquid biopsy technology may pave the way for improved diagnostic and prognostic tools in the treatment of MBC.

## 3. Histological Markers

Histological molecular markers are currently the standard for the classification of malignant breast neoplasms. As previously indicated, the main markers are currently estrogen receptors and HER2. One of the most relevant reviews in classifying molecular types came from Parise et al., who evaluated 61,309 patients, of which 80% were estrogen receptor-positive, 23% were HER2-positive, and 13% were not positive for any markers, which represent the triple negatives [76].

This molecular classification has also allowed it to be used as a predictive factor for response to different targeted therapies. In the case of metastatic disease, there is a discordance between the expression of biomarkers of the metastatic lesion and the original lesion in the breast, which has been studied by several authors. De Dueñas et al. [37] evaluated 184 patients and found alterations in both HER 2 and estrogen/progesterone receptor expression in secondary tumors when compared to the primary tumor. Thus, a significant proportion of patients with metastatic disease have a dissonance between the original tumor and the metastasis, which led to changes in the therapeutic regimen received in 31% of such patients, therefore highlighting the importance of re-evaluating metastatic disease [37]. In another study evaluating 289 patients with MBC, Amir et al. found a molecular discordance rate between the original breast lesion and metastases of 12.6% for estrogen receptors, 31.2% for progesterone receptors, and 5.5% for HER2 receptors, leading to a change in management based on molecular alterations in 14% of all patients [38].

The first discoveries of estrogen receptors and molecular markers led to a revolution with the FDA approval in 1978 of the first selective estrogen receptor modulator, tamoxifen [39,40]. Subsequently, from a pharmacological point of view, selective estrogen receptor downregulation such as fulvestrant has been developed, as well as other estrogen deprivation therapies such as anastrozole or GnRH analogs such as goserelin. In women with hormone-sensitive metastatic neoplasia, the addition of cyclin CDK4 and CDK6 inhibitors to hormone therapy has brought about a real change in the prognosis of patients with metastatic neoplasia. One of the most relevant pool analyses is that carried out by Gao et al., which included 4200 patients from 7 studies demonstrating an improvement in progression-free survival of 6.9 months in the group that combined estrogen inhibitors with cyclin inhibitors [41].

In recent years, the discovery of different molecular alterations has broadened the therapeutic arsenal, leading to an attempt to standardize the usefulness of the different therapies available. This is why we must highlight the usefulness of The Scale of Clinical Actionability for Molecular Targets. The Scale of Clinical Actionability for Molecular Targets (ESCAT) is a collaborative project initiated by the ESMO Precision Medicine and Translational Research Working Group. It was published in 2018 in the Annals of Oncology, a framework for classifying genomic alterations as targets for cancer precision medicine. This scale classifies molecular targets numerically, from I to V, based on the available evidence supporting their value as clinical targets [77,78,79,80,81,82,83].

For example, an ESCAT I means that the alteration–drug combination is associated with better outcomes in clinical trials; therefore, they are ready for routine use. An ESCAT V means that the alteration–drug match is associated with an objective response but without clinically meaningful benefit.

In breast cancer, the main biomarkers (endocrine receptors, HER2, PD-L1, *BRCA*, PIK3CA...) present an ESCAT I, which provides sustainable evidence to be able to use them in clinical practice [83].

The implementation of a harmonized vocabulary would help clinicians to prioritize cancer genomic abnormalities when interpreting genomic reports and would facilitate communication between academia, the pharmaceutical industry, healthcare professionals, and patients.

### 3.1. HER 2. The First Steps of Targeted Therapy in MBC

Another of the most relevant markers, as we have previously indicated, is HER2, described by Slamon et al. in 1987 [42]. HER2 is a transmembrane tyrosine kinase receptor that plays a key role in cell proliferation and survival. HER2 amplification or overexpression occurs in approximately 15–20% of breast cancers and is associated with aggressive tumor characteristics and poor prognosis [42]. Since then, different lines of research have led to the development of different drugs targeting HER2, such as trastuzumab, pertuzumab, and lapatinib. The development of HER2-targeted therapies has revolutionized the treatment of HER2-positive MBC. The introduction of trastuzumab, a monoclonal antibody targeting HER2, marked an important milestone in the treatment of HER2-positive MBC. In pivotal clinical trials, trastuzumab combined with chemotherapy demonstrated significant improvements in response rates, progression-free survival (PFS), and overall survival (OS) compared with chemotherapy alone [42]. Despite the success of trastuzumab, treatment resistance remains a major clinical challenge. The development of new HER2-targeted agents, such as pertuzumab, ado-trastuzumab emtansine (T-DM1), and neratinib, has expanded the therapeutic options for patients with HER2-positive MBC. The CLEOPATRA trial demonstrated that the addition of pertuzumab, a monoclonal antibody directed against HER2, to trastuzumab and docetaxel, significantly improved PFS and OS in first-line treatment of HER2-positive MBC [43]. In patients with HER2-positive MBC who progressed on trastuzumab, the EMILIA trial demonstrated that T-DM1, an antibody–drug conjugate, significantly improved PFS and OS compared with lapatinib plus capecitabine [44]. In this sense, the TH3RESA trial further confirmed the clinical benefit of T-DM1 in patients who had received multiple lines of anti-HER2 therapy [45]. The NALA trial demonstrated that neratinib, an irreversible pan-HER tyrosine kinase inhibitor, combined with capecitabine, improved PFS compared with lapatinib plus capecitabine in patients with HER2-positive MBC who had received at least two prior anti-HER2 regimens [46]. Despite significant advances in HER2-targeted therapies, resistance and disease progression remain inevitable for most patients with HER2-positive MBC. New therapeutic strategies, such as immune checkpoint inhibitors, bispecific antibodies, and novel antibody–drug conjugates, are being actively investigated to further improve outcomes in this patient population. In recent years, the usefulness of immunoconjugated drugs such as trastuzumab–deruxtecan has been studied. Multicenter clinical trials such as DESTINY Breast 03 observed a progression-free survival of 28.8 months [47]. In some tumors, the expression of HER2 is lower than in HER2-positive tumors; these tumors are called HER2-low (immunohistochemistry [IHC] 1+ or IHC 2+/in situ hybridization [ISH]-negative). In general, HER2-low tumors have a better prognosis than HER2-positive tumors and may respond to specific targeted treatments and other anticancer therapies. The FDA has approved Fam-trastuzumab deruxtecan for patients with unresectable or metastatic HER2-low breast cancer who have recurred within six months of receiving adjuvant chemotherapy. This option would also be useful in patients with positive hormone receptors refractory to endocrine therapy [55].

### 3.2. PD-L1. The Role of Immunotherapy in MBC

In another direction, the importance of PD-L1 has been observed in recent years in triple-negative tumors. PD-L1, a transmembrane protein, binds to its receptor, programmed cell death 1 (PD-1), which is expressed on the surface of T cells, leading to inhibition of T cell activation and promotes immune evasion [48]. PD-L1 expression has been described in various types of cancer, including breast cancer. In breast cancer, PD-L1 expression has been found to vary between subtypes, with higher expression levels in triple-negative (TNBC) and HER2-positive breast cancer subtypes [49]. Immunotherapy targeting the PD-1/PD-L1 axis has shown promise in several malignancies, including non-small-cell lung cancer, melanoma, and renal cell carcinoma. In MBC, the efficacy of PD-1/PD-L1 inhibitors has been investigated primarily in unresectable locally advanced or metastatic TNBC, where higher levels of PD-L1 expression are observed [49].

The phase III IMpassion130 trial demonstrated that the addition of atezolizumab, a PD-L1 inhibitor, to nab-paclitaxel significantly improved PFS in untreated TNBC patients with PD-L1-positive tumors [50]. In the atezolizumab in combination with chemotherapy treatment group, the median PFS was 7.5 months, compared to 5.0 months in the chemotherapy-alone treatment group. The tumor response rate was 53% in the atezolizumab treatment group, compared with 33% in the chemotherapy-only treatment group [50]. In addition, KEYNOTE-355 evaluated the efficacy of pembrolizumab, a PD-1 inhibitor, combined with chemotherapy in patients with PD-L1-positive TNBC, showing that in the 3-week pembrolizumab treatment group, the PFS median was 9.7 months, compared to 5.6 months in the chemotherapy-only treatment group, with a tumor response rate of 53.3% in the pembrolizumab treatment group, compared to 44.2% in the chemotherapy-only treatment group [51].

Despite these promising results, in breast cancer, checkpoint inhibitors have so far not shown as clear and consistent efficacy as in other types of cancer, such as melanoma and non-small cell lung cancer, since not all PD-L1-positive TNBC patients respond to immunotherapy; therefore, identification of reliable biomarkers to predict response remains an active area of research [52].

### 3.3. PIK3CA. The Role of Kinases in MBC

Mutations in PIK3CA are found in approximately 30–40% of breast cancers, with a higher prevalence in HR+/HER2− tumors [52]. PIK3CA is a gene that encodes for the p110α catalytic subunit of the enzyme phosphatidylinositol-4,5-bisphosphate 3-kinase (PI3K). PI3K plays a critical role in cellular signaling pathways that regulate various cellular functions, including cell growth, proliferation, differentiation, motility, survival, and intracellular trafficking. PIK3CA is part of the larger PI3K/AKT/mTOR pathway, which is one of the most frequently dysregulated signaling pathways in human cancers. Mutations in the PIK3CA gene can lead to the overactivation of the PI3K pathway, resulting in increased cellular proliferation and reduced apoptosis, contributing to tumorigenesis. The most common mutations in breast cancer are located in the helical (exon 9) and kinase (exon 20) domains, with the hotspots being E545K, E542K, and H1047R, accounting for approximately 80% of all PIK3CA mutations [53]. Targeting the PI3K pathway has emerged as a promising therapeutic strategy for breast cancer patients with PIK3CA mutations. The development of isoform-specific PI3K inhibitors, such as alpelisib, has demonstrated significant clinical benefits in the treatment of HR+/HER2− MBC patients with PIK3CA mutations. The phase III SOLAR-1 trial demonstrated that the addition of alpelisib to fulvestrant significantly improved progression-free survival (PFS) in patients with HR+/HER2− MBC with PIK3CA mutations [54]. Based on these results, alpelisib was approved by the FDA for the treatment of HR+/HER2− MBC with PIK3CA mutations in 2019.

### 3.4. The Uncommon Markers. Tumor-Infiltrating Lymphocytes, Androgen Receptors, and BCL2

Other biomarkers that have been studied include tumor-infiltrating lymphocytes (TILs). TILs are immune cells that infiltrate the tumor microenvironment, and their presence has been associated with improved clinical outcomes in certain breast cancer subtypes. In particular, higher TIL levels have been correlated with better prognosis and response to neoadjuvant chemotherapy in TNBC and HER2-positive breast cancer [84,85]. Further studies are needed to elucidate the role of TILs in MBC and to determine their potential as a predictive marker for immunotherapy.

In this line, androgen receptor (AR), which is a nuclear hormone receptor, has been identified as a potential therapeutic target in breast cancer, particularly in the luminal androgen receptor (LAR) subtype of TNBC. The presence of AR has been associated with a more favorable prognosis in ER-positive breast cancer [55]. In TNBC, AR-targeting agents such as enzalutamide and bicalutamide have shown preliminary antitumor activity [86].

Another biomarker that is important to note in breast cancer is BCL2. BCL2 is an anti-apoptotic protein that plays a key role in cell survival and has been implicated in tumor progression and resistance to therapy. High BCL2 expression has been associated with favorable outcomes in ER-positive breast cancer [87]. However, the prognostic and therapeutic implications of BCL2 in MBC remain to be elucidated. The development of BCL2 inhibitors, such as venetoclax, may offer a new therapeutic avenue for BCL2-positive breast cancers [88].

### 3.5. ADH and ALDH in MBC and Their Potential Use

The molecular mechanisms underlying breast cancer progression and metastasis remain an area of active research. Recent studies have highlighted the roles of alcohol dehydrogenase (ADH) and aldehyde dehydrogenase (ALDH) isoenzymes in breast cancer development and progression. The ADH family comprises multiple isoenzymes involved in the oxidation of alcohols to aldehydes. ADH1B and ADH1C isoenzymes have been implicated in breast cancer. Overexpression of these isoenzymes has been associated with increased cancer aggressiveness and poorer prognosis. They contribute to the regulation of cell proliferation, migration, and invasion, essential processes in metastasis [56,57,89]. The ALDH family, particularly the ALDH1A subfamily, catalyzes the oxidation of aldehydes to carboxylic acids. Elevated ALDH1A1 expression has been associated with breast cancer stem cells (CSCs), which contribute to tumor initiation, progression, and metastasis. ALDH1A1+ breast cancer cells exhibit a more aggressive phenotype, increased migration, invasion, and resistance to chemotherapy [58]. ADH and ALDH isoenzymes may modulate various signaling pathways, such as the Wnt/β-catenin, Notch, and PI3K/Akt pathways, which are known to regulate cell proliferation, survival, and metastasis. Additionally, reactive aldehydes and reactive oxygen species (ROS) generated through ADH and ALDH activity can induce DNA damage and promote tumor growth [59]. Their potential use as therapeutic targets is related to the inhibition of ADH and ALDH isoenzymes, which could reduce the metastatic potential of breast cancer cells and increase their sensitivity to chemotherapy [90]. These isoenzymes represent promising therapeutic targets in the management of breast cancer. Further research is needed to develop specific inhibitors and assess their efficacy and safety in preclinical and clinical settings.

### 3.6. BRCA Mutations in MBC. Their Role in Genetic and Hereditary Breast Cancer

In recent years, the importance of different genetic markers in breast cancer has also been highlighted, with the importance of *BRCA1* and *BRCA2* standing out. *BRCA1* and *BRCA2* are tumor suppressor genes involved in DNA repair by homologous recombination. Germline mutations in *BRCA1/2* significantly increase the risk of developing breast and ovarian cancer [91]. BRCA-associated breast cancers often have distinctive clinicopathological features, such as higher grade, triple-negative phenotype, and increased genomic instability [60]. The deficiency in homologous recombination repair in *BRCA*-mutated tumors has led to the development of targeted therapies, such as poly (ADP-ribose) polymerase (PARP) inhibitors. PARP inhibitors exploit synthetic lethality by targeting base excision repair, leading to the accumulation of unrepaired DNA damage and, ultimately, cell death in *BRCA*-mutated cancer cells [61]. In patients with germline *BRCA*-mutated MBC, the phase III OlympiAD trial demonstrated that olaparib, a PARP inhibitor, significantly improved progression-free survival (PFS) compared with standard chemotherapy [62]. Another phase III trial, EMBRACA, showed that talazoparib, a potent PARP inhibitor, significantly improved PFS in patients with germline *BRCA*-mutated MBC compared with standard chemotherapy [63]. Based on these results, both olaparib and talazoparib have been approved for the treatment of germline *BRCA*-mutated, HER2-negative MBC. In addition, platinum-based chemotherapy has shown promising activity in *BRCA*-associated breast cancers due to its high sensitivity to DNA damage [64]. However, further research is needed to define the optimal sequence and combination of targeted therapies and chemotherapy in this patient population.

In conclusion, histological markers play a pivotal role in predicting prognosis and guiding therapeutic decisions in MBC. Continued research into emerging markers and the development of new targeted therapies hold promise for improving outcomes for patients with MBC.

## 4. MicroRNA. Implications in Diagnosis and Prognosis

MicroRNAs are small non-coding RNA molecules with a length of ~20 nucleotides that regulate the post-transcriptional expression of genes that may be related to cell differentiation, proliferation, and apoptosis processes by promoting or suppressing the expression of a gene after transcription. A miRNA molecule regulates the post-transcriptional expression of up to 200 different genes, and its study may expand the understanding of the underlying pathophysiology of the metastatic process [65]. In relation to breast cancer, numerous implications of miRNAs in diverse pathways such as cellular proliferation, metastatic invasion, and therapy resistance, have been highlighted through the upregulation or downregulation of either tumor suppressor genes or oncogenes. [66] In this line, Asaga et al. demonstrated the usefulness of miR-21 in differentiating advanced metastatic breast neoplasia in 102 patients versus 20 healthy controls, obtaining AUC curves of 0.721 [67]. Similarly, Schawarzenbach evaluated in 102 patients how elevated levels of miR 214 were associated with increased lymphatic infiltration and metastatic disease as well as being able to differentiate benign breast neoplasms and absence of breast pathology [68]. We should highlight the work of Eichelser et al., who observed how the serological elevation of miR-17 and miR-155 is accompanied by greater metastatic involvement in 120 patients with breast cancer [69]. Authors such as Zhao et al. evaluated 122 breast cancer patients showing how an elevation of miR-10b is accompanied by metastatic bone disease [70].

The importance of microRNAs has also been evaluated in terms of their prognostic usefulness, which may allow better classification of tumors with a higher risk of recurrence and invasion. In this regard, authors such as Chen et al. evaluated in 159 patients how overexpression of miR 191 5p and downregulation of miR-214-3p, miR 451a, and miR-489 were associated with worse median survival and disease-free survival [71]. Along these lines, Cascione et al. evaluated 173 women with triple-negative tumors where miR-16, miR-155, or miR-374a overregulation was associated with a better median survival of 83 months, while mir 125b downregulation was associated with a worse prognosis, with a median survival of 69 months [72]. These results are complemented by the line of Gasparini et al. in 173 patients with triple-negative breast neoplasms where overregulation of miR-155 and miR-493 was associated with better prognosis, with a median survival of 82 months, while downregulation of miR-30e and miR-27 was associated with a worse median survival of 75.5 months [73]. Other authors, such as Jayasingam et al., have shown the importance of the combination of 11 microRNAs (miR-21, miR-24-2, miR-125a, miR-221, miR-22, miR-501, miR-365b, miR-660, miR-146a, let-7b, and miR-31) in 1063 patients with breast cancer as the presence of both under- and over-expression is associated with worse prognosis and worse median survival regardless of the type of molecular marker or tumor subtype [74]. On the other hand, authors such as Chen et al. have shown in 450 patients with triple-negative breast cancer that patients with higher miR-223 expression have better median survival and progression-free survival compared to those with lower expression of this microRNA [92]. Therefore, we can see how different authors have demonstrated the usefulness of different microRNAs in the prognosis of advanced breast cancer, which makes it possible to anticipate tumors with a worse prognosis and can be used as markers of risk of progression.

## 5. Circulating Tumor Cells. Liquid Biopsy

The concept of circulating tumor cells (CTCs) refers to cancer cells that break away from a primary tumor and enter the bloodstream or lymphatic system and can metastasize to distant organs, which may contribute to disease progression. By definition, they are usually cells expressing epithelial cell adhesion proteins (EpCAM), cytokeratin 8, 18, and/or 19 positive, with an intracellular nucleus, no CD45 expression, and at least 4 μm in size. Although rare, CTCs may be found in non-tumor inflammatory conditions such as Crohn’s disease or endometriosis to a lesser extent than in tumor processes [93].

CTCs are an important object of study in cancer research, as their presence and number in the blood can provide information about prognosis and response to treatment. In addition, their molecular analysis can provide information on the genetic and molecular characteristics of the primary tumor, which may be useful in the selection of specific targeted therapies [94]. However, there are challenges in the detection and analysis of CTCs, such as variability in the number and characteristics of the detected cells, as well as the lack of standardized standards and methods in their analysis. Despite these challenges, CTCs remain an important area of cancer research.

Multiple methods have been studied for the detection of CTCs; currently, the detection method approved by the FDA is based on the detection of EpCAM/cytokeratin-expressing cells in blood using antibodies through the CellSearch platform. Other methods such as EpCAM positive immunoselection, leukocyte negative immunoselection, filtration, immunomagnetism, electrophoresis, or flow cytometry have also proven useful but are currently not approved by the FDA, as they are based on complex techniques [95]. In reference to liquid biopsy, the main limitations of this technique are mainly based on sample collection and processing techniques, as CTCs can become fragile and cannot be properly processed [96]. In addition, these techniques are costly and technically complex, requiring a support laboratory that not all hospitals have. Therefore, despite the immense benefits of CTC detection, these techniques are accompanied by limitations that may restrict their use in actual clinical practice. Numerous studies have established a directly proportional relationship between disease burden and the level of CTCs in the blood. Thus, using the CellSerch system, a threshold count ≥1 CTC/7.5 mL of blood is set for non-metastatic disease and a count ≥5 CTCs/7.5 mL of blood for metastatic disease [75].

The relevance of CTCs has already been described in prostate, breast, and colon cancer, where their presence is accompanied by a worse prognosis and higher recurrence rates after chemotherapy or surgery [77,78]. Regarding its clinical application in breast cancer, it has proven useful in both early and MBC [79]. With regard to early breast cancer, in those patients who are candidates for adjuvant chemotherapy, pre-detection of CTCs aims to determine whether a tumor has initiated micrometastatic spread to distant sites and potentially monitor early tumor response to systemic treatment. A CTC detection rate of around 20–22% before the initiation of neoadjuvant chemotherapy has been reported in some studies [80]. In MBC, the detection of CTCs in blood is an independent predictor of therapeutic efficacy as well as a prognostic marker. In patients who persist with a high level of CTCs or higher than pre-treatment levels, it suggests resistance to chemotherapy and could, therefore, guide the decision to initiate a particular cancer treatment [81]. Cristofanilli et al. examined the role of CTC counts in predicting prognosis, treatment response, and disease spread in MBC. The researchers looked at 36 patients with stage III and 203 patients with stage IV breast cancer. They found that CTC counts were much higher in stage IV patients, with an average of 62.2 cells/7.5 mL of blood compared to 14.5 cells in stage III, and that within each group, higher CTC counts predicted worse outcomes [82]. CTCs have been suggested as a prognostic tool to monitor metastasis or chemotherapy efficacy; however, according to ASCO expert panels, the role of CTCs in monitoring treatment response remains controversial and should not be used to influence treatment decisions in metastatic disease at this time [81].

In this line, the importance of circulating tumor deoxyribonucleic acid (ctDNA) has been proven. ctDNA is a fragment of DNA released by tumor cells into the bloodstream. Detection of ctDNA in the blood can provide important information about the presence, tumor burden, and molecular characteristics of cancer, including specific gene alterations [97].

One of the most important alterations that can be detected in ctDNA is the estrogen receptor alpha (ESR1) mutation. ESR1 mutations are common in patients with advanced breast cancer previously treated with hormonal therapy and are associated with resistance to hormonal treatments. Therefore, the detection of ESR1 mutations in ctDNA may be useful in making therapeutic decisions. Some of the more common ESR1 mutations that can be detected in ctDNA include *D538G*, *Y537S*, *Y537C*, and *E380Q*. Studies have shown that patients with these mutations are less likely to respond to standard hormone treatments and may benefit from alternative therapies, such as CDK4/6 inhibitors or inhibitors of the PI3K signaling pathway [98,99].

In summary, the detection of ctDNA and ESR1 alterations in ctDNA can provide valuable information on tumor burden and resistance to hormonal treatments in patients with advanced breast cancer. The results can influence treatment decisions and help personalize treatment for each individual patient.

## 6. Current Therapeutic Decisions Based on the Different Biomarkers in Metastatic Breast Cancer

As we have seen, the identification of different biomarkers has allowed us to improve the approach to metastatic breast cancer as well as to better understand the underlying pathophysiology of this disease and to demonstrate its importance in the diagnosis. The identification of these biomarkers allows for a more personalized approach to MBC treatment. Biomarker-guided therapy selection can improve patient outcomes by matching patients with the most effective treatments, sparing them from unnecessary toxicity and reducing healthcare costs.

**Figure 1:**
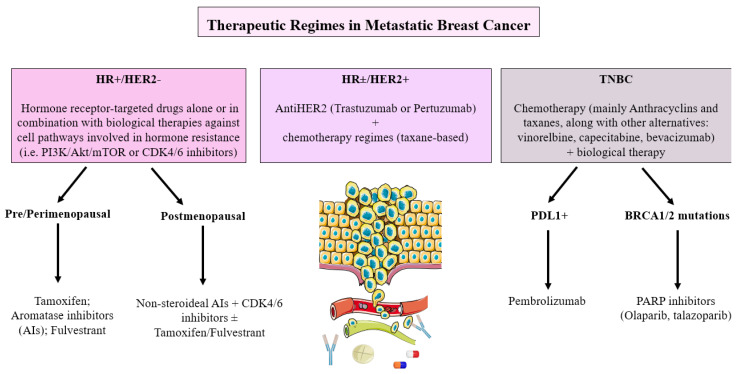
Therapeutic regimes in metastatic breast cancer.

The current implications in therapeutic decisions are evidenced in different clinical guidelines of various oncological societies that standardize treatment to improve overall survival and quality of life of patients, in addition to reducing the toxicities of systemic chemotherapies that have been used classically. According to the expression of the different biomarkers, we can resume the actual treatment of metastatic breast cancer and it can be summarized in Figure 1.

### 6.1. Hormone Receptor-Positive/HER2-Negative Metastatic Breast Cancer

The management of these tumors, called “luminal”, focuses mainly on endocrine therapy (ET), blocking the estrogen signal, which varies depending on the menopausal state. However, with the new advances in biomarkers and resistance to ET, new therapeutic targets have been developed since the latest guidelines. Hormone receptor-targeted drugs are commonly used as single agents or in combination with therapies directed against pathways involved in hormone resistance, such as mTOR inhibitors (imTOR) such as everolimus (PIK3/AKT/mTOR), and CDK4/6 inhibitors, such as palbociclib, ribociclib and abemaciclib (cell cycle pathway) [55]. Among the estrogen receptor inhibitors, there are Selective Estrogen Receptor Modulators (SERM), such as tamoxifen or toremifen, and Selective Estrogen Receptor Downregulators (SERD), such as fulvestrant [86].

To know which ET to use, several aspects have to be taken into account, including whether they have received neoadjuvant ET, disease-free interval, response to previous ET, burden of disease and symptoms, menopausal status, comorbidities, patient preferences, costs, and availability. However, the optimal sequence of ET is uncertain.

First-line options available in pre- and peri-menopausal women with ovarian function suppression/ablation and post-menopausal women include aromatase inhibitors (AI), tamoxifen, fulvestrant, or combinations such as AI or fulvestrant with CDK inhibitors [76]. The combination of an AI/fulvestrant with a CDK 4/6 inhibitor increases progression-free survival (PFS) by 10 months compared to ET monotherapy; however, it increases toxicity. This option is preferred if there is no contraindication [41].

Non-steroidal AI, such as anastrozole or letrozole, and steroidal AI, such as exemestane, are superior to tamoxifen, with no differences in efficacy between the three AI [40]. In patients who have not previously received ET and without visceral disease, fulvestrant, at a dose of 500mg, has a higher PFS than anastrozole (18 vs. 13 months) with no differences in overall survival (OS) [39,40]. In the second line, it will depend on the drug used in the previous lines. For those patients who have received prior AI, fulvestrant 500mg could be administered. Another option would be to change the type of AI from steroidal to non-steroidal or vice versa, but the benefits obtained are modest [39]. In patients with no prior exposure to CDK4/6 inhibitors, the combination of CDK4/6 inhibitors together with fulvestrant has been shown to be more useful than the use of fulvestrant alone. Another option to take into account is the combination of mTOR, such as everolimus with AI, where an increase in PFS has been shown compared to AI in monotherapy [53]. Taking all these data into account, a CDK4/6 inhibitor should be added to ET as soon as possible, as the side effect profile is more tolerable than other therapies, such as mTOR, where toxicity is more severe. ET should be continued until the disease progresses or to relevant toxicity. Currently, there are no validated predictive markers to identify those women who might benefit from treatment with CDK inhibitors or mTOR [76].

In this sense, resistance to CDK4/6 inhibitors is a common problem in patients with advanced breast cancer treated with these drugs. Massive sequencing or NGS studies have identified several molecular alterations that may contribute to resistance to CDK4/6 inhibitors, including RB1 mutations, CCND1 amplification, CDK6 overexpression, and activation of the PI3K signaling pathway [22].

This resistance can be intrinsic or acquired. Intrinsic resistance may be due to activation of the PI3K signaling pathway, overexpression of CDK6, inactivation of RB1, or amplification of CCND1. On the other hand, acquired resistance can arise due to acquired mutations in RB1 or CDK4/6, activation of other cell survival signaling pathways, or clonal selection of resistant tumor cells [41,52]. To overcome resistance to CDK4/6 inhibitors, several therapeutic strategies have been developed. One of the most promising strategies is the combination of CDK4/6 inhibitors with other antitumor agents, such as inhibitors of the PI3K signaling pathway, PARP inhibitors, and endocrine therapy. The combination of CDK4/6 inhibitors and inhibitors of the PI3K signaling pathway has been shown to be particularly effective in preclinical models and clinical trials [53].

In addition, new CDK4/6 inhibitors are being evaluated that may be more effective in overcoming resistance to existing CDK4/6 inhibitors. These new CDK4/6 inhibitors may have different selectivity profiles, mechanisms of action, and spectrums of activity, which may be beneficial in addressing tumor heterogeneity and acquired resistance [54].

### 6.2. HER2 Positive Metastatic Breast Cancer

The first-line treatment approach, regardless of hormone receptor positivity or negativity, should include a combination of taxane-based and anti-HER-2 chemotherapy, such as trastuzumab or pertuzumab. In the phase III CLEOPATRA study, it was shown that there is greater PFS and OS in targeted therapy with double anti-HER2 blockade (trastuzumab and pertuzumab) associated with chemotherapy [44]. In the second line, according to the phase III EMILIA study, chemotherapy based on T-DM1 would be the most recommended regimen, although it should be taken into account that the data on efficacy in patients previously exposed to pertuzumab are limited. It demonstrated superiority in PFS and OS compared to other therapies such as lapatinib or capecitabine [44,45]. Chemotherapy regimens that have not been used in the first or second lines should be required in subsequent lines [46]. Regarding the duration of treatment, it was individualized for each case. In general, it is recommended that the chemotherapy last a minimum of 6 months, depending on toxicity and/or tumor progression. When the chemotherapy regimen is stopped or changed, anti-HER2 therapy should be continued even in subsequent lines, as the available data suggest that the benefit is maintained at least until the third line [43].

### 6.3. Triple-Negative Metastatic Breast Cancer

This type of tumor is characterized by the absence of estrogen, progesterone receptors, and HER2 expression. Chemotherapy is the standard treatment. The regimen used will depend on multiple factors, so it must be individualized. However, in general, the use of monotherapy agents is preferred, leaving the use of combination therapies to patients with more aggressive, symptomatic, or life-threatening diseases. In the first line, chemotherapy regimens based on anthracyclines and taxanes are considered the best option. If there is resistance or secondary toxicity to anthracyclines, taxane-based therapy could be considered as a single agent [46,47]. In patients previously treated with taxanes or anthracyclines, other options, such as vinorelbine and capecitabine, are available [46]. In some patients with aggressive disease, being HER2 negative, adding bevacizumab, a humanized anti-VEGF monoclonal antibody, can be considered. It is important to highlight that there is no limit to the number of lines of therapy in patients with metastatic triple-negative breast cancer as long as an adequate quality of life is maintained. Some of the preferred available drugs include capecitabine, vinorelbine, eribulin, or others such as nab-paclitaxel, gemcitabine, or platinum derivatives [50]. The determination of PDL-1 is useful in these patients since, according to the results of the KEYNOTE-355 trial, adding pembrolizumab to the chemotherapy regimen in those patients with high % PDL-1 expression improves overall survival compared to the use of chemotherapy alone [48,49,51]. The optimal duration of treatment is not established, but it is generally given until progression or unacceptable toxicity.

### 6.4. BRCA1/BRCA2 Metastatic Breast Cancer

For patients with germline *BRCA* mutations in metastatic breast cancer, poly ADP ribose polymerase (iPARP) inhibitors such as olaparib or talazoparib are useful treatment options [58]. Specifically, olaparib has been approved by the European Medicines Agency (EMA) after progression to chemotherapy without presenting platinum-resistant disease. Longer PFS (approximately 3 months), as well as a higher response rate with a good side effect profile and better quality of life, have been demonstrated compared to other standard therapies [24,25].

Thus, we can see the clinical implications of the various biomarkers in the selection of a wide range of therapies and how decisions on the clinical approach are based on the molecular biology of each subtype of metastatic breast cancer.

## 7. Conclusions

**Figure 2:**
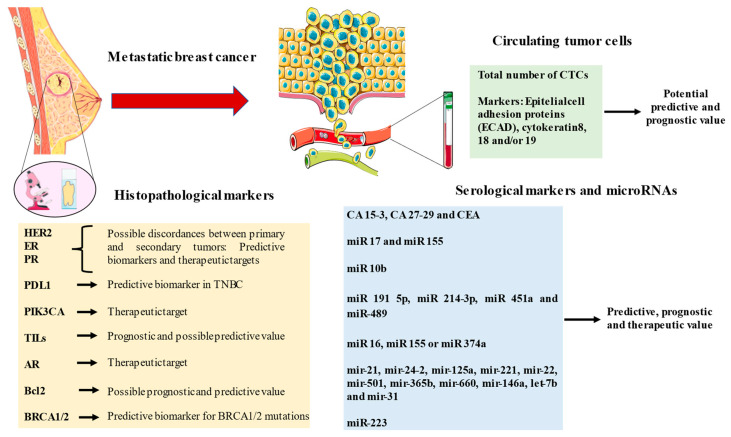
A summary of the main biomarkers explored in metastatic breast cancer.

Biomarkers have been a real revolution in the diagnosis and approach to breast cancer. In Figure 2, the main biomarkers explored in this manuscript are collected. Despite their promising uses, the monitoring of the disease throughout its natural course is still based on imaging tests and serological markers that are sometimes too complex for the clinician to interpret. Therefore, we note that there are a wide variety of markers that can expand the diagnostic arsenal in patients with MBC.

## Figures and Tables

**Table 1:** Summary of the main utilities of different biomarkers in breast cancer.

Biomarker	Utility	Reference
CA 15-3/CA 27-29	Serological marker to monitor disease progression and/or response to a given treatment	[28,29,30,31,32,33,34,35,36]
HER2 and Hormonal Receptors	Molecular classification of breast cancer in luminal A, luminal B, Her 2, and triple-negative breast cancer.	[37,38,39,40,41,42,43,44,45,46,47,48,49,50]
PDL1	Expression of more than >1% defines sensitivity to PDL1 inhibitors in triple-negative breast cancer.	[48,49,50,51]
PIK3CA	Usefulness of PI3K inhibitors such as alpelisib in hormone-positive receptor/HER2-negative metastatic breast cancer.	[52,53,54]
Androgen receptor	More favorable prognosis in ER-positive breast cancer.	[55]
BRCA1/2	Sensitivity to targeted therapies (PARP inhibitor) such as olaparib and talazoparib in patients with BRCA mutations.	[56,57,58,59]
MicroRNA (miR-21, miR- miR 17, miR 155, of miR-10b, miR 191 5p, miR 214-3p, miR 451a and miR-489, miR 16, miR 155 or miR 374a)	Impact in prognosis and diagnosis of metastatic breast cancer.	[60,61,62,63,64,65,66,67,68,69,70,71,72,73]
Circulating tumor cells.	Count ≥ 5 CTCs/7.5 mL of blood is related to metastatic disease, and >62.2 cells/7.5 mL is related to a worse prognosis in MBC.	[74,75]

## Data Availability

Not applicable.
